# Innovative Approaches to Inclusive Design for Technology to Support Aging With Disability: Examples From TechSAge

**DOI:** 10.1093/geroni/igaa057.2017

**Published:** 2020-12-16

**Authors:** Tracy Mitzner, Anne Ordway

## Abstract

Technology research and development often exclude older adults with disabilities from participating in the design process. As a result, technologies may not be useful or usable by older adults with diverse abilities. This symposium, featuring projects at the TechSAge Rehabilitation Engineering Research Center, highlights ongoing efforts toward inclusive design, representing unique approaches to engage older adults with disabilities and their stakeholders in the research and development of technology supports. First, Mitzner et al., will describe the development of an online, group Tai Chi intervention, and the integral involvement of older adults with mobility disabilities, the exercise program developers, and technology partner in all steps of the process. Exploring the potential of voice-activated assistants, like Amazon Alexa, to support health management activities of older adults with mobility disabilities, Kadlyak et al. will present findings from a needs assessment of the target population and user testing in the lab and home environments. Koon et al. will present findings from a subject matter expert interview study with caregivers and medical professionals designed to identify the scope of activity challenges among people aging with long-term mobility and sensory disabilities that should be explored in more depth through our future interview study with the target population. Sanford et al., will describe a student design competition and hackathon that incorporates immersive experiences with people aging with disabilities to inspire innovative design concepts that respond to the needs of real people. NIDILRR Project Officer, Anne Ordway, will serve as the discussant.

